# Effects of fermented diet on reproductive performance and fecal microbiota for late-gestation sows

**DOI:** 10.3389/fnut.2025.1502193

**Published:** 2025-05-02

**Authors:** Hai Sheng, Chaoqi Liu, Zhentian Li, Ping Wang, Lijun Wang, Sanjun Jin, Xinxin Li, Lin Yuan, Juan Chang, Qingqiang Yin, Qun Zhu, Fushan Lu

**Affiliations:** ^1^College of Animal Science and Technology, Henan Agricultural University, Zhengzhou, China; ^2^Institute of Animal Husbandry and Veterinary Medicine, Henan Academy of Agricultural Sciences, Zhengzhou, China; ^3^Henan Delin Biological Product Co. Ltd., Xinxiang, China; ^4^Henan Puai Feed Co. Ltd., Zhoukou, China

**Keywords:** fermented diet, pregnant sows, litter performance, nutrient digestibility, serum biochemical parameters, fecal microbiota

## Abstract

The late-gestation period is a key stage in the reproductive cycle of sows, which is related to the health of sows and the fetal development. In order to increase sow reproduction in late gestation, the fermented diet was produced and applied in this study. A total of 24 sows in the third pregnancy were divided into four groups on the 80th day gestation, 6 sows in each group. Group A was a basal diet served as the control group; groups B, C and D were added with 5, 10 and 15% fermented diet, respectively. The experiment was 35 days (from the 80th day gestation until parturition). The results showed that the sows in group D exhibited the best reproductive performance. Compared to group A, litter weight in group D was increased by 24.91% (*P* **<** 0.05). Additionally, crude protein digestibility in groups B, C, and D was significantly higher than that in group A (*P* **<** 0.05). Serum levels of TC, TG, IL-6, TNF-α, ROS and MDA in group D were significantly decreased, while serum levels of TP, ALB, GLB, IL-10, IgA, IgG and GSH-Px were significantly increased, compared with group A (*P* < 0.05). 16S rRNA analysis indicated that the relative abundances of *Bacteroidota*, *Turicibacter* and *norank_f__Muribaculaceae* in group D were significantly increased, while the relative abundances of *Proteobacteria*, *Escherichia-Shigella* and *Aerococcus* were significantly decreased, compared with group A (*P* < 0.05). The abundances of other genera between groups A and D were insignificantly different (*P* > 0.05). In conclusion, the fermented diet addition in the basal diet of late-gestation sows could adjust the balance of gastrointestinal microbiota as well as enhance protein digestibility, anti-oxidative capacity and immunity for improving their reproductive performance.

## 1 Introduction

The late-gestation period is a critical phase in the reproductive cycle of sows, which significantly influences both sow health and fetal development. The previous studies have demonstrated that 35% of the increase in fetal weight occurs during the final 10 days of gestation (1), and the nutritional status of sows in the late-gestation period directly affects their mammary gland development and lactation ability (2). During the late stage of gestation, sows confront a myriad of challenges. This period is characterized by profound transformations in physiology, metabolic processes, immune responses and gut microbiota of sows (2). These alterations intensify the oxidative stress and inflammation, thereby directly impacting the sow’s overall productivity (3, 4). Therefore, feeding strategies in the late-gestation period are crucial to influence sow reproductive performance.

Currently, swine nutritionists are delving into the potential feed additives and feeding strategies to alleviate the above problems for improving sow reproductive performance (5). Among the effective strategies, the inclusion of fermented diet can regulate gut microbiota and immune status as well as enhance antioxidant capabilities, thereby improving the health and reproductive performance of sows (6, 7). Previous studies reported that dietary supplementation with fermented diet in sows from late gestation to lactation improved nutrient digestibility and enhanced piglet performance (8, 9). However, most studies focus on the lactation stage of sows, while the study on the late-gestation stage is limited.

The feed microbial fermentation process involves the transformation of feed substrates by using artificially added microorganisms (10), which not only enhances the activity of beneficial microbes but also significantly decomposes anti-nutritional factors, increases the concentrations of enzymes and metabolites, and inhibits proliferation of harmful bacteria (11, 12). The beneficial microorganisms frequently utilized in the production of fermented diet encompass lactic acid bacteria, *Bacillus*, yeasts and some molds (13). Lactobacilli metabolize substances to produce organic acids such as lactic acid, acetic acid and propionic acid, which can effectively lower pH value in the gut, thereby inhibiting the growth of harmful bacteria (14). Additionally, lactic acid bacteria exhibit a remarkable ability to neutralize free radicals (15). Previous researches reported that adding lactobacilli-fermented diet into the diets of livestock and poultry notably enhanced the growth performance (16), and fortified the resistance to oxidative stress (17, 18). *Bacillus* secretes digestive enzymes, antimicrobial peptides and bacteriocins, making it an ideal strain for fermentation (19–21). Another report showed that incorporating *Bacillus licheniformis* into the feed effectively mitigated the incidence of diarrhea in weaned piglets and promoted intestinal health (22). Additionally, the soybean hull fermented by *Bacillus velezensis* was found to enhance intestinal antioxidant capacity and downregulated the expression of pro-inflammatory gene in broilers (23). During the fermentation process, *Saccharomyces cerevisiae* plays a pivotal role in the biological transformation that produces alcohol, organic acids and anti-inflammatory factors, enhancing the flavor and aroma of the feed (24–26). The further study showed that the metabolic byproducts of *Saccharomyces cerevisiae* enhanced the productive performance of broilers, mitigated inflammatory responses, and bolstered their immune system (27). Therefore, it is inferred that the functions of lactic acid bacteria, *Bacillus* and *Saccharomyces* will help to improve the health status of pregnant sows during the late-gestation period. In addition, many important biochemical reactions comprise the fermentation process, and these can’t be completed by a single microorganism. It requires co-fermentation of many microbial strains to fully utilize the synergistic advantages of each microorganism (12, 13). However, the production of the fermented diet by lactic acid bacteria, *Bacillus* and *Saccharomyces* composite and its application in late-gestation sows have not been reported. Therefore, this study was designed to investigate the effects of synergistically fermented diet using multiple microbial strains on litter performance, antioxidant capacity, immune status and gut microbiota in sows during late gestation, aiming to provide an effective strategy for improving sow reproductive performance.

## 2 Materials and methods

### 2.1 Animal ethics statement

All the procedures were approved by the Institutional Animal Care and Use Committee in Henan Agricultural University (Grant No. HENAU-2023-018).

### 2.2 The preparation of fermented diet

The fermented diet used in this experiment was prepared by fermenting the basal diet with four species of microbes such as *Lacticaseibacillus casei* (CGMCC 1.2884), *Lactiplantibacillus pentosus* (GDMCC 1.505), *Bacillus velezensis* (GDMCC 1.3870) and *Saccharomyces cerevisiae* (GDMCC 2.167), which was added in the basal diet at concentrations of 1 × 10^6^ CFU/g, 1 × 10^6^ CFU/g, 1 × 10^6^ CFU/g and 1 × 10^5^ CFU/g, respectively, based on the previous results in our laboratory. The fermentation process was carried out by mixing the basal diet with 40% water, and fermented at 37 ± 2°C for 3 days. *L. casein*, *L. pentosus*, *B. velezensis*, and *S. cerevisiae* were kindly provided by Henan Delin Bioproducts Co. Ltd., Xinxiang, China. The basal diet was composed of 65.51% corn meal, 8.70% soybean meal, 21.70% wheat bran, 1.30% limestone, 1.00% CaHPO_4_, 0.45% NaCl, 0.18% lysine, 0.03% methionine, 0.13% threonine and 1.00% premix.

### 2.3 Animals diets and managements

In this experiment, 24 sows with almost the same backfat thickness in the third gestation were selected and divided into four groups on the 80th gestation, 6 sows in each group, each sow was kept in one individual farrowing crate. Group A was a basal diet served as the control group; groups B, C, and D were added with 5, 10, and 15% fermented diet based on air-dry matter to replace equivalent amounts of basal diet, respectively. The counts of *Bacillus velezensis* in the diets of groups A, B, C and D were 0, 4.71 × 10^7^, 9.42 × 10^7^ and 1.41 × 10^8^ CFU/kg, respectively. The counts of *Saccharomyces cerevisiae* in the diets of groups A, B, C and D were 0, 1.32 × 10^8^, 2.64 × 10^8^ and 3.97 × 10^8^ CFU/kg, respectively. The counts of lactobacilli including *Lacticaseibacillus casei* and *Lactiplantibacillus pentosus*, in the diets of groups A, B, C and D were 0, 1.10 × 10^11^, 2.20 × 10^11^ and 3.31 × 10^11^ CFU/kg, respectively. The experimental period was 35 days, from the 80th gestation until parturition. The pregnant sows were restricted to meals, and fed twice daily at 6 am and 2 pm. Water was given *ad libitum*. The room temperature was maintained at 25 ± 2°C with the relative humidity of 55%–60%. The sows were managed and immunized according to the farm’s standard protocols. The basal diet was designed according to the standards (NRC 2012). The feed composition and nutrient levels were listed in Table 1. The nutrient levels of fermented diet were presented in Table 2.

**TABLE 1:** Basal diet composition and nutritional content (air-dry basis, %).

Item	Content	Item	Content
**Diet compositions**			
Corn	65.51	Lysine	0.18
Soybean meal	8.70	Methionine	0.03
Wheat bran	21.70	Threonine	0.13
Limestone	1.30	Premix^1^	1.00
CaHPO_4_	1.00	Total	100
NaCl	0.45		
**Nutrient levels^2^**			
Crude protein	13.04	Calcium	0.76
Digestible energy (MJ/Kg)	12.70	Total phosphorus	0.62
Ether extract	3.30	Lysine	0.60
Crude fiber	4.30	Threonine	0.22

^1^The premix provided the following per Kg of the diet: VA 11150 IU; VB_2_ 8.0 mg; VB_12_ 30 μg; VD_3_ 2210 IU; VE 66 IU; VK 1.42 mg; biotin 0.44 mg; DD-pantothenic acid 23.6 mg; folic acid 1.59 mg; niacin 44.1 mg; choline 350 mg; Cu 20 mg; Fe 145 mg; Zn 145 mg; Mn 60.2 mg; Se 0.35 mg; I 1.26 mg.

^2^Crude protein, ether extract, calcium and total phosphorus contents were measured, while the other nutrient contents were calculated.

**TABLE 2:** Nutrient levels in basal diet before and after microbial fermentation (air-dry basis, %).

Nutrient levels	The basal diet	The fermented basal diet
Crude protein	13.04	13.29
Ether extract	3.30	3.97
Calcium	0.76	0.73
Total phosphorus	0.62	0.60

### 2.4 Sample measurements

At the start and end of the experiment, the backfat thickness (BFT) at the last rib (P2 point: 6.5 cm from the midline of the last rib) of the sows was measured using an ultrasound backfat scanner (PIGLOG105, SFK-Technology, Herlev, Denmark). On the day of farrowing, the total litter size, number of live birth, still birth and litter weight for each sow were recorded. The live birth rate (number of live birth / total litter size) and the average body weight of piglet (litter weight / number of live birth) were calculated.

Fecal samples were collected from four sows in each group for three days at the end of gestation, respectively. The 3-day fecal sample was mixed and stored at −20°C for further analysis. The fecal samples were dried at 65°C and ground to determine nutrient digestibility. Crude protein, ether extract, calcium and phosphorus contents in diets and feces were measured with Kjeldahl, ether extract, potassium permanganate (KMnO_4_), and ammonium molybdate [(NH_4_)_6_Mo_7_O_24_] protocols, respectively. The ash insoluble in 4 N hydrochloric acid was used as an indicator to calculate nutrient digestibility. Nutrient digestibility (%) = 100–[(indicator content in diet / nutrient content in diet) × (nutrient content in feces / indicator content in feces) × 100].

On the 110th day of gestation, about 10 mL blood samples were collected from the precaval veins of 4 sows in each group. After the blood was kept at room temperature for 3 h, the serum was collected using a transferpettor and stored in a centrifuge tube at −20°C for further analysis. The concentrations of aspartate aminotransferase (AST), alanine aminotransferase (ALT), alkaline phosphatase (ALP), lactic dehydrogenase (LDH), glucose (GLU), total cholesterol (TC), triglycerides (TG), urea nitrogen (UN), total protein (TP) and albumin (ALB) contents in the serum were analyzed using the automatic biochemical analyzer (Chemray 800, BIOBASE, Jinan, China). The content of globulin (GLB) was obtained by subtracting albumin from total protein. The concentrations of interleukin-6 (IL-6), interleukin-10 (IL-10), tumor necrosis factor-alpha (TNF-α), immunoglobulin G (IgG), immunoglobulin A (IgA), immunoglobulin M (IgM), glutathione peroxidase (GSH-Px), superoxide dismutase (SOD), catalase (CAT), reactive oxygen species (ROS) malondialdehyde (MDA) and hydrogen peroxide (H_2_O_2_) contents in serum were quantified using ELISA kits from the Shanghai Enzyme-linked Biotechnology Co., Ltd. Shanghai, China.

### 2.5 DNA extraction and 16S rRNA sequencing for fecal microbiota analysis

To study the effect of fermented diet on fecal microbiota, fecal samples were collected without contamination from 4 sows in groups A and D on the day of parturition. The total genomic DNA was extracted from fecal sample using Soil DNA Kit (Omega Biotek, Norcross, GA, USA) according to the manufacturer’s instructions. The final DNA concentration and purity were investigated by NanoDrop 2000 UV–vis spectrophotometer (Thermo Scientific, Wilmington, NC, USA), and DNA quality was checked by 1% agarose gel electrophoresis. The V3–V4 region of 16S rRNA was amplified with 338F up-stream primer (5′-ACTCCTACGGGAGGCAGCAG-3′) and 806R down-stream primer (5′-GGACTACHVGGGTWTCTAAT-3′) by PCR (GeneAmp 9700, ABI, USA). The PCR amplification program was set as follows: initial denaturation at 95°C for 3 min; 28 cycles of 95°C for 30 s, annealing at 55°C for 30 s, elongation at 72°C for 45 s; and a final extension at 72°C for 10 min. PCR reaction was carried out in 20 μL reaction mixture containing 10 ng template DNA, 4 μL 5 × FastPfu buffer, 2 μL 2.5 mM dNTPs, 0.8 μL each primer (5 μM), and 0.4 μL FastPfu polymerase (2.5 U/μL). The amplified PCR products were extracted by 2% agarose gel, purified by the AxyPrep DNA Gel Extraction Kit (Axygen Biosciences, Union City, CA, USA), and further quantified using QuantiFluorTM-ST [Promega (Beijing) Biotech Co., Ltd. Beijing, China] according to the manufacturer’s instructions. The purified amplification products were pooled in equimolar amounts and sequenced on an Illumina MiSeq platform (Illumina, San Diego, CA, USA).

The 16S rRNA gene amplicon data have been deposited in the National Center for Biotechnology Information Sequence Read Archive (accession number: PRJNA1248386).

### 2.6 Bioinformatics analysis of sequencing data

The operational taxonomic units (OTUs) clustering analysis was conducted on sequences with a similarity of 97% using Uparse software (version 11).^1^ Each sequence was annotated for species classification using the RDP classifier (version 2.13),^2^ and species comparison was performed against the Silva 16S rRNA database (version 138). The analysis of α-diversity indices (Ace and Chao) and β-diversity (principal component analysis, PCA) was performed through the Majorbio cloud platform.^3^ The Wilcoxon rank-sum test was used to identify differential microbes, and the Spearman correlation test assessed the relationships between fecal microbiota and environmental parameters.

### 2.7 Statistical analysis

Statistical analyses were performed using SPSS Statistics Software (version 25.0, IBM, New York, NY, USA). Data were evaluated by one-way ANOVA, followed by Duncan’s test for comparative analysis. The results were presented as means ± SEM. Statistical significance was established at a level of *P* < 0.05.

## 3 Results

### 3.1 Litter performance and nutrient digestibility of sows affected by fermented diet

Table 3 showed that litter weight in group D was significantly increased by 24.91% (*P* < 0.05), compared with group A. There were no significant differences (*P* > 0.05) among 4 groups in total litter size, number of live birth, number of still birth, live birth rate, average body weight of piglet and backfat thickness (BFT); however, the live birth rate and average body weight of piglets showed an upward trend in groups B, C and D, compared to group A.

**TABLE 3:** Effect of different proportions of fermented diet on litter performance of sows (*n* = 6).

Items	A^1^	B^2^	C^3^	D^4^
Total litter size	16.83 ± 1.94	16.00 ± 2.97	15.83 ± 3.92	16.67 ± 2.50
Number of live birth	14.67 ± 1.94	14.50 ± 2.97	14.33 ± 3.92	15.67 ± 2.50
Number of still birth	2.17 ± 0.75	1.50 ± 1.05	1.50 ± 1.05	1.00 ± 0.63
Live birth rate (%)	87.11 ± 4.37	91.29 ± 5.26	91.13 ± 5.38	93.96 ± 4.03
Average body weight of piglets (kg)	1.21 ± 0.21	1.26 ± 0.25	1.49 ± 0.36	1.41 ± 0.16
Litter weight (kg)	17.54 ± 2.49^b^	18.02 ± 3.16^b^	20.49 ± 2.12^ab^	21.91 ± 2.77^a^
BFT on the 80th day of gestation (mm)^5^	17.00 ± 2.37	17.83 ± 2.48	18.67 ± 3.08	18.67 ± 3.08
BFT at parturition (mm)	21.17 ± 3.87	22.17 ± 2.64	22.17 ± 2.64	21.67 ± 2.73

^1^A: basal diet.

^2^B: 5% fermented diet addition in the basal diet.

^3^C: 10% fermented diet addition in the basal diet.

^4^D: 15% fermented diet addition in the basal diet.

^5^BFT: backfat thickness.

^a,b^The different lowercase letters in the same rows indicate significant difference (*P* < 0.05), while the same or without lowercase letters in the same rows indicate insignificant difference (*P* > 0.05).

Table 4 revealed that crude protein digestibility in 3 groups added with fermented diet was significantly higher than that in group A (*P* < 0.05); however, there were no significant differences for other nutrient digestibility among 4 groups (*P* > 0.05).

**TABLE 4:** Effects of different proportions of fermented diet on nutrient digestibility of sows (%) (*n* = 4).

Items	A^1^	B^2^	C^3^	D^4^
Crude protein	80.30 ± 1.27^b^	83.26 ± 1.47^a^	83.20 ± 2.10^a^	85.76 ± 1.73^a^
Ether extract	72.75 ± 3.07	75.19 ± 3.68	75.70 ± 3.88	76.73 ± 2.53
Calcium	53.93 ± 2.54	51.74 ± 1.51	53.09 ± 2.76	54.65 ± 2.60
Phosphorus	47.71 ± 1.53	49.72 ± 1.13	48.15 ± 1.63	48.83 ± 1.70

^1^A: basal diet.

^2^B: 5% fermented diet addition in the basal diet.

^3^C: 10% fermented diet addition in the basal diet.

^4^D: 15% fermented diet addition in the basal diet.

^a,b^The different lowercase letters in the same rows indicate significant difference (*P* < 0.05), while the same or without lowercase letters in the same rows indicate insignificant difference (*P* > 0.05).

### 3.2 Effect of fermented diet on serum biochemical parameters of sows

Table 5 showed that serum TC content in group A was significantly higher than that in other groups (*P* < 0.05), and serum TG content in group A was significantly higher than that in groups C and D (*P* < 0.05). Serum TP, ALB and GLB contents in group D were significantly higher than those in groups A and B (*P* < 0.05), indicating that 15% fermented diet addition in the diet of pregnant sows could significantly improve immunity, protein and lipid metabolisms. In addition, there were no significant differences for other serum biochemical parameters among 4 groups (*P* > 0.05).

**TABLE 5:** Effects of fermented diet on serum biochemical parameters of sows (*n* = 4).

Items	A^1^	B^2^	C^3^	D^4^
AST (U/L)	37.91 ± 9.37	39.33 ± 13.65	36.84 ± 9.54	31.00 ± 2.05
ALT (U/L)	61.43 ± 12.95	57.83 ± 6.32	57.33 ± 7.37	53.98 ± 5.15
ALP (U/L)	45.25 ± 11.44	45.25 ± 11.44	42.25 ± 4.79	43.25 ± 16.98
LDH (U/L)	326.75 ± 90.03	405.00 ± 77.05	324.50 ± 43.49	347.00 ± 25.50
GLU (mmol/L)	3.50 ± 0.41	3.45 ± 0.85	3.55 ± 0.44	3.64 ± 0.83
TC (mmol/L)	3.29 ± 0.21^a^	2.88 ± 0.21^b^	2.62 ± 0.13^bc^	2.58 ± 0.19^c^
TG (mmol/L)	0.59 ± 0.04^a^	0.53 ± 0.07^ab^	0.46 ± 0.07^b^	0.48 ± 0.07^b^
UN (mmol/L)	7.02 ± 0.5	6.74 ± 0.41	6.89 ± 0.74	6.92 ± 0.36
TP (g/L)	57.90 ± 1.83^b^	60.25 ± 2.23^b^	64.43 ± 3.13^a^	67.70 ± 1.94^a^
ALB (g/L)	35.18 ± 3.08^b^	36.43 ± 1.02^b^	38.2 ± 2.2^ab^	40.05 ± 0.85^a^
GLB (g/L)	22.73 ± 3.30^b^	23.83 ± 1.51^b^	26.23 ± 1.77^ab^	27.65 ± 2.37^a^

AST, aspartate aminotransferase; ALT, alanine aminotransferase; ALP, alkaline phosphatase; LDH, lactic dehydrogenase; GLU, glucose; TC, total cholesterol; TG, triglycerides; UN, urea nitrogen; TP, total protein; ALB, Albumin; GLB, globulin.

^1^A: basal diet.

^2^B: 5% fermented diet addition in the basal diet.

^3^C: 10% fermented diet addition in the basal diet.

^4^D: 15% fermented diet addition in the basal diet.

^a,b,c^The different lowercase letters in the same rows indicate significant difference (*P* < 0.05), while the same or without lowercase letters in the same rows indicate insignificant difference (*P* > 0.05).

### 3.3 Serum inflammatory factors, immunoglobulins and antioxidant indices of sows affected by fermented diet

**FIGURE 1:**
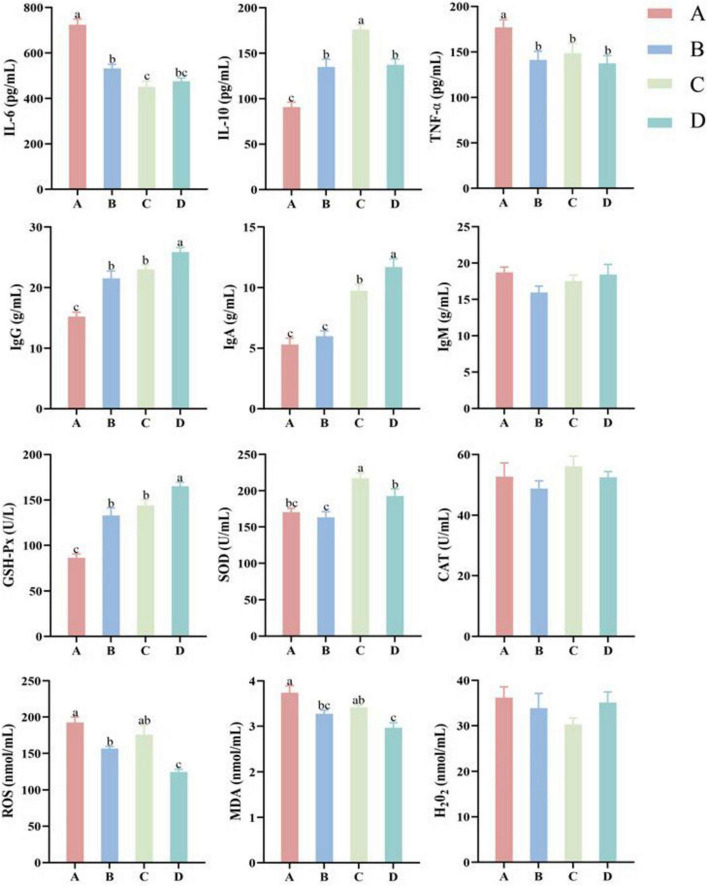
Effect of different proportions of fermented diet on inflammatory factors, immunoglobulins, and antioxidant indices in sow serum (*n* = 4). IL-6, interleukin-6; IL-10, interleukin-10; TNF-α, tumor necrosis factor-alpha; IgG, immunoglobulin G; IgA, immunoglobulin A; IgM, immunoglobulin M; GSH-Px, glutathione peroxidase; SOD, superoxide dismutase; CAT, catalase; ROS, reactive oxygen species; MDA, malondialdehyde; H_2_O_2_, hydrogen peroxide. A: basal diet; B-D: 5, 10, and 15% fermented diet addition in the basal diet, respectively. ^a,b,c^The different lowercase letters on each bar indicate significant difference (*P* < 0.05), while the same or without lowercase letters on each bar indicate insignificant difference (*P* > 0.05).

Figure 1 indicated that serum IgA, IgG and GSH-Px contents in group D were significantly higher than those in other groups (*P* < 0.05), while serum ROS and MDA contents in group D were significantly lower than those in other groups (*P* < 0.05), indicating that 15% fermented diet addition in the diet of pregnant sows could significantly improve immunity and antioxidative capacity. Serum IL-6 and TNF-α contents in group A were significantly higher than those in other groups (*P* < 0.05), while serum IL-10, IgG and GSH-Px contents in group A were significantly lower than those in other groups (*P* < 0.05). Serum SOD content in group C was significantly higher than that in other groups (*P* < 0.05). In addition, ROS and MDA contents in group A were significantly higher than those in groups B and D (*P* < 0.05). There were no significant differences for serum IgM, CAT and H_2_O_2_ contents among 4 groups (*P* > 0.05).

### 3.4 Fecal microbiota adjusted by fermented diet

**FIGURE 2:**
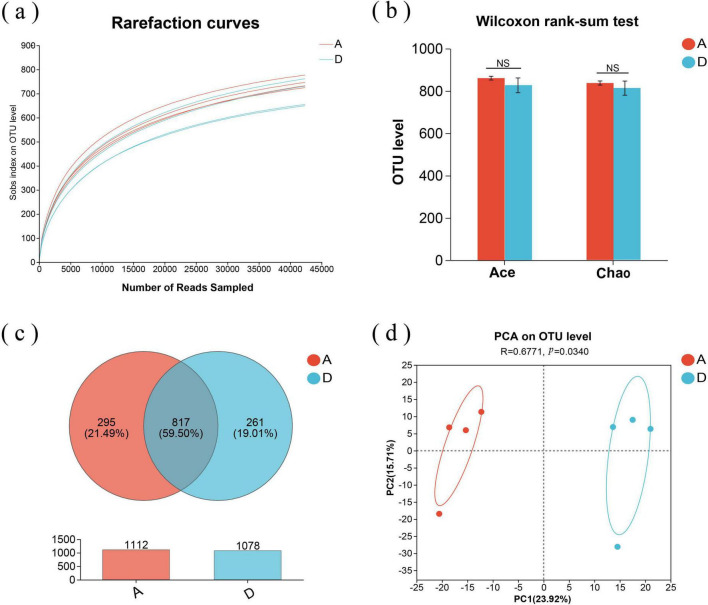
The fecal microbiota of sows in groups A and D. **(a)** Rarefaction curves of OTUs at 97% similarity for every sample (based on the Sobs index); **(b)** Microbial α-diversity indexes such as Ace and Chao; **(c)** Venn diagrams for OTUs; **(d)** PCA; NS, no significant difference. A: basal diet; D: 15% fermented diet addition in the basal diet.

The V3-V4 regions of 16S rRNA were sequenced from eight fecal samples (four samples for groups A and D) using the Illumina MiSeq high-throughput sequencing platform. Figure 2a indicated that the sequencing depth was sufficient to cover the microbial diversity in each sample. The microbial alpha diversity indices in Figure 2b revealed insignificant differences for Ace and Chao indices between both groups (*P* > 0.05), suggesting that feeding fermented diet did not change the alpha diversity of fecal microbiota for pregnant sows. Figure 2c showed that there were a total of 817 common operational taxonomic units (OTUs) shared between both groups, accounting for 59.50%; 295 unique OTUs in group A, accounting for 21.49%; 261 unique OTUs in group D, accounting for 19.01%. Principal component analysis (PCA) depicted in Figure 2d revealed significant differences in the compositions of microbial communities between both groups (*P* < 0.05).

### 3.5 Effect of fermented diet on fecal bacterial communities of pregnant sows

**FIGURE 3:**
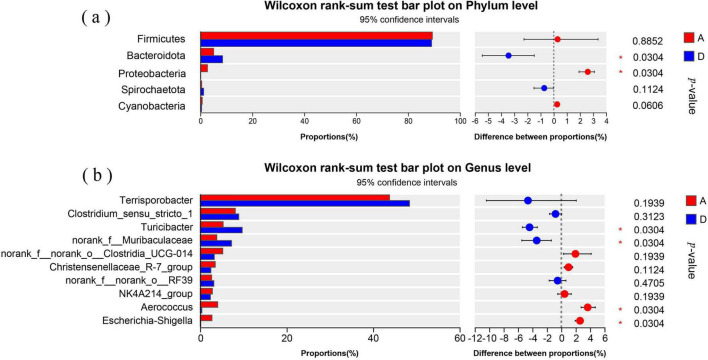
Differences in the fecal bacterial communities of sows between groups A and D. **(a)** Differences in the relative abundances of top 5 phyla. **(b)** differences in the relative abundances of top 10 genera. A: basal diet; D: 15% fermented diet addition in the basal diet.

Figure 3a showed the relative abundances of top 5 bacterial phyla, in which *Firmicutes* was dominant in both groups, accounting for 89.39% in group A and 89.09% in group D (*P* > 0.05). *Bacteroidota* abundance in group D was significantly higher than that in group A (*P* < 0.05), while *Proteobacteria* abundance in group D was significantly lower than that in group A (*P* < 0.05). The abundances of *Spirochaetota* and *Cyanobacteria* in both groups were insignificantly different (*P* > 0.05).

Figure 3b displayed the relative abundances of top 10 genera, in which *Terrisporobacter* was dominant in both groups, accounting for 43.78% in group A and 48.40% in group D (*P* > 0.05). The relative abundances of *Turicibacter* and *norank_f__Muribaculaceae* in group D were significantly higher than that in group A (*P* < 0.05), while *Escherichia-Shigella* and *Aerococcus* abundances in group D were significantly lower than that in group A (*P* < 0.05). The abundances of other genera in both groups were insignificantly different (*P* > 0.05).

### 3.6 Correlation analysis between fecal microbiota and environmental factors

**FIGURE 4:**
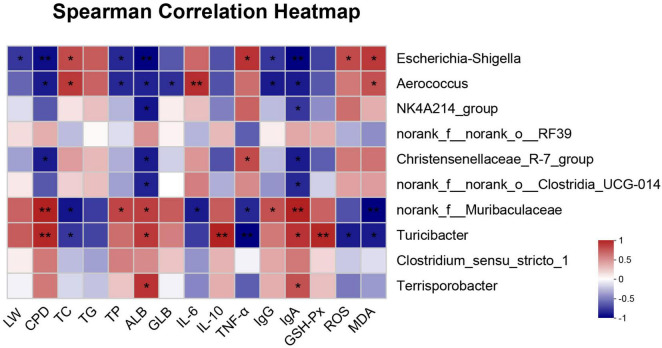
Spearman correlation analysis between top 10 genera and environmental factors. The depth of colors represent the strength of correlation. Red indicates positive correlation, whereas blue indicates negative correlation. LW, litter weight; CPD, crude protein digestibility; TC, total cholesterol; TG, triglycerides; TP, total protein; ALB, albumin; GLB, globulin; IL-6, interleukin-6; IL-10, interleukin-10; TNF-α, tumor necrosis factor-alpha; IgG, immunoglobulin G; IgA, immunoglobulin A; GSH-Px, glutathione peroxidase; SOD, superoxide dismutase; ROS, reactive oxygen species; MDA, malondialdehyde. *P < 0.05, **P < 0.01.

The Spearman correlation analysis was conducted between the environmental factors and fecal top 10 genera, which was directly reflected through a heatmap (Figure 4). The threshold |R| > 0.5 was considered as being correlated. The results indicated that *Turicibacter* was positively correlated with crude protein digestibility (CPD) and serum ALB, IL-10, IgA GSH-Px levels (*P* < 0.05), but negatively correlated with serum TC, TNF-α, ROS and MDA levels (*P* < 0.05). *norank_f__Muribaculaceae* was positively correlated with CPD and serum TP, ALB, IgG, IgA levels (*P* < 0.05), while negatively correlated with serum TC, IL-6, TNF-α and MDA levels (*P* < 0.05). *Aerococcus* was positively correlated with serum TC, IL-6 and MDA levels (*P* < 0.05), while negatively correlated with CPD and serum TP, ALB, GLB, IgG, IgA levels (*P* < 0.05). *Escherichia-Shigella* was positively correlated with serum TC, TNF-α, ROS and MDA levels (*P* < 0.05), but negatively correlated with litter weight (LW) and serum TP, ALB, IgG, IgA levels (*P* < 0.05).

## 4 Discussion

The physiological and nutritional status of sows will affect live birth rate, litter weight and piglet weight (2). The birth weight influences lifetime performance, as it affects postnatal survival, weaning weight and market weight (28). Previous studies have demonstrated that the inclusion of *Saccharomyces*, *Bacillus* and organic acids in the diets of pregnant sows can enhance the live birth rate (29–31). In this study, supplementing 15% fermented diet in the late stages of pregnancy decreased stillbirths, increased the live birth rate and birth litter weight. The reason may be that the fermented biomass is rich in beneficial substances such as probiotics, organic acids, amino acids, peptides and enzymes, which can improve the utilization rate of nutrients (32), consequently promoting the health of sows and embryo development. Moreover, the addition of 5, 10, and 15% fermented diet in this study improved the digestibility of crude protein, consistent with previous research (33). A study on the co-fermentation of *Bacillus velezensis* and *Lactiplantibacillus plantarum* with soybean meal showed reductions in anti-nutritional factors such as soy glycinin and β-conglycinin, while increasing the content of amino acids and TCA-soluble proteins (34). This may be the reason for increasing crude protein digestibility. Research has found that over two-thirds of fetal weight gain in sows occurs during the late stage of pregnancy, in which crude protein and amino acid intake played a crucial role in this process (35). In this study, high crude protein digestibility caused by the fermented diet will provide enough protein for sows, which may be the reason to increase litter birth weight.

Serum albumin, the most abundant plasma protein, plays essential roles in maintaining colloid osmotic pressure, transporting substances, scavenging free radicals, and regulating immune responses (36). Serum globulins are also a major component of serum total protein, usually calculated by subtracting serum albumin from total protein. Serum globulins include globulins, fibrinogen, C-reactive protein, interleukins, and all non-albumin proteins, which play vital roles in the immune system by producing antibodies to combat pathogens and regulating immune responses (37). This study showed that 15% fermented diet addition significantly increased the levels of serum albumin, globulin and total protein, inferring that the fermented diet displayed beneficial effects on the health of sows (38). The elevated concentrations of amino acids, peptides and probiotics in the fermented diet may contribute to its active functions (39, 40). In late pregnancy of sows, hyperlipidemia is common, which can increase the risk of metabolic disorders, unfavorable for the litter performance of sows (41). Generally, high levels of TC and TG in serum indicate the elevated lipid levels. In this study, the fermented diet addition reduced serum TC and TG contents, which is beneficial to sows and litter performance. It has been reported that fermentation by lactic acid bacteria increased the yield of soluble dietary fiber (SDF) derived from proso millet bran and improved its cholesterol adsorption capacity (42). Another study reported that adding high-level SDF from wheat bran in the diet effectively reduced TC and TG levels compared to low-level SDF, thereby preventing abnormalities in lipid metabolism and improving the litter performance of sows (43). Therefore, the decrease in serum TC and TG levels observed with the inclusion of fermented diet can be attributed to the elevated content of SDF derived from wheat bran and the improvement of its cholesterol adsorption capacity via microbial fermentation.

During pregnancy, a dysregulation in the network of inflammatory cytokines might have led to adverse pregnancy outcomes such as spontaneous miscarriage, preterm birth and intrauterine growth retardation (44). The maternal immune response is spontaneously regulated to reduce body’s rejection of the fetus, and this regulation was primarily achieved through changes in cytokine secretion (45). IL-6 is a characteristic manifestation of the systemic inflammatory response syndrome (46). IL-10 is an immunosuppressive cytokine to induce immune tolerance, playing a role in inhibiting uterine motility during late pregnancy to avoid premature birth (47, 48). TNF-α is a potent pro-inflammatory cytokine that negatively impacts embryonic development in rats, mice, and humans (47, 49). Immunoglobulins protect the body from external bacterial and viral invasions, and their levels are important indicators for assessing the humoral immune response in animals (50). IgG is considered to be associated with protecting the fetus from maternal immune attacks during the late stages of pregnancy (47). IgA plays a role as the first line of immune defense on the body’s mucosal surfaces and in bodily fluids (50). Additionally, IgA is capable of crossing the placenta into the bodies of piglets via active transport mechanisms and could also be present in the colostrum (8). In this experiment, feeding sows with 15% fermented diet in the late stage of pregnancy significantly reduced the levels of IL-6 and TNF-α in serum, and significantly increased the levels of IL-10, IgG and IgA, thereby enhancing the immune status of sows. It was reported that feeding sows with 10% fermented feed during lactation significantly increased the levels of IgG and IL-10 in serum, and significantly reduced TNF-α levels (7). Another report indicated that feeding sows with 15% fermented feed during lactation significantly increased the content of IgA in the milk (9). Previous studies have demonstrated that *Lactiplantibacillus plantarum*, *Saccharomyces cerevisiae*, *Bacillus subtilis* and their metabolites such as short-chain fatty acids can increase the levels of immune in the host’s blood (7, 51, 52). The main reason was that probiotics and metabolites in the fermented diet could serve as standalone antigens that activated the intestinal mucosa, epithelial cells and associated lymphoid tissues as well as promoted lymphocyte proliferation, ultimately boosting the body’s immune response (17, 53).

Pregnancy is considered a state of oxidative stress (54), particularly during the late stage, when rapid fetal growth occurs alongside substantial alterations in lipid and glucose metabolism. The increase in oxidative reactions during maternal cellular metabolism further intensifies the level of oxidative stress (55). High stillbirth rate during the late stages of pregnancy in sows is positively correlated with high state of oxidative stress (56). This is primarily manifested by the elevated levels of ROS and MDA within the body. ROS represents the total of free radicals and peroxides such as OH−, O− and H_2_O_2_. MDA levels could reflect the extent of lipid peroxidation and indirectly indicate the degree of damage caused by free radicals in the body (6). The body’s antioxidant defense system including SOD and GSH-Px, plays a crucial role in scavenging reactive oxygen species (17). Adding 6% fermented soybean meal in diet for lactating sows significantly reduced serum MDA levels and significantly increased serum GSH-Px concentrations, while supplementation with 4% fermented soybean meal significantly elevated serum SOD concentrations (6). Supplementing the diet of sows in late pregnancy with *Saccharomyces cerevisiae* fermentation products significantly increased the concentration of GSH-Px in serum (57). This study found that adding 15% fermented diet in late pregnancy of sows significantly increased the content of GSH-Px in the serum and significantly reduced the levels of ROS and MDA; 10% fermented diet addition significantly increased the serum SOD levels, corresponding with the previous researches. It was reported that lactobacilli and their metabolites could activate the Nrf2 pathway in animal liver and red blood cells, thereby promoting the transcription of SOD and GSH-Px genes and enhancing the body’s antioxidant capabilities (58).

In late pregnancy, metabolic disorders and immune imbalances may lead to disruptions in the gut microbiota, with the community structure and composition exhibiting characteristics similar to disease-associated dysbiosis (59). An increase in the circulating concentration of bacterial lipopolysaccharides (LPS) can cause metabolic endotoxemia, which subsequently induces a series of inflammatory responses, ultimately affecting the reproductive capacity of sows (60). Fermented feed plays a crucial role in modulating the composition of the sow’s gut microbiota and maintaining a healthy gastrointestinal ecosystem by alleviating excessive inflammatory responses to intestinal pathogens (7, 61). *Firmicutes* and *Bacteroidetes* are the most predominant component within the sow fecal microbiota (62). In this experiment, the fecal microbiota was primarily made up of *Firmicutes* and *Bacteroidetes*, consistent with the previous report (62, 63). Furthermore, feeding fermented diet significantly increased the relative abundances of *Bacteroidetes*, *Turicibacter* and *norank_f__Muribaculaceae*, while concurrently decreasing the relative abundances of *Proteobacteria*, *Escherichia-Shigella*, and *Aerococcus*. *Bacteroidota* is capable of producing propionate and acetate, which are essential for both the host and the maintenance of gut microbiota homeostasis (64). Moreover, *Bacteroidota* could break down macromolecular compounds in the intestinal tract, enhancing the digestion and absorption of proteins, lipids and polysaccharides (32). The ratio of *Firmicutes* to *Bacteroidota* (F/B) is considered as an important indicator of energy metabolism in mammals and is typically associated with energy deposition, in which a lower value indicates less energy storage (65). In this experiment, the fermented diet increased *Bacteroidota* abundance and decreased F/B ratio, suggesting low energy storage in the body, which may be beneficial to fetal development and high litter weight. *Turicibacter* is an important member in gut microbiota to reduce the levels of TC and TG in serum as well as the weight of adipose tissue (66). Additionally, it can reduce levels of TNF-α and MDA in serum, thereby alleviating oxidative stress and inflammatory responses (67). In this study, *Turicibacter* abundance was significantly increased by fermented diet addition, which exhibited negative correlations with serum TNF-α, ROS, MDA and TC levels, and a positive correlation with serum GSH-Px activity. These findings suggest that the fermented diet can reduce serum TC concentration and enhance antioxidant capabilities for sows, maybe through *Turicibacter* regulation. *norank_f__Muribaculaceae* is enriched in a healthy gut environment (68). It was reported that *norank_f__Muribaculaceae* was positively correlated with the expression of genes related to the intestinal barrier function in pigs (69). Another report showed that feeding fermented wheat bran improved the immune levels of fattening pigs and increased the relative abundance of *norank_f__Muribaculaceae* in the intestine (70). In this study, the Spearman correlation analysis showed that *norank_f__Muribaculaceae* was significantly negatively correlated with serum IL-6 and TNF-α levels, and significantly positively correlated with serum IgA, IgG, CPD, TP and ALB levels. Therefore, it can be inferred that the fermented diet can increase immune responses in sows, maybe due to increasing gut *norank_f__Muribaculaceae* abundance. Additionally, *norank_f__Muribaculaceae* also showed a strong negative correlation with serum TC level, suggesting that *norank_f__Muribaculaceae* plays an important role in alleviating dyslipidemia, corresponding with the previous study (71). *Proteobacteria* is a tiny component within a balanced gut microbiota. However, it has been indicated that an increase in the diagnostic microbial signature associated with imbalanced gut microbiota, intestinal inflammation and epithelial dysfunction can be attributed to the expansion of *Proteobacteria* (72). *Proteobacteria* includes many common opportunistic pathogens such as the well-known *Escherichia-Shigella*. *Proteobacteria* and *Escherichia-Shigella* can generate LPS (73). Once bacterial LPS enters the circulation through a compromised gut barrier, the concentration of bacterial endotoxins in the circulation will be increased, potentially acting as a mediator of inflammation to cause metabolic endotoxemia, oxidative stress and inflammation (74, 75). In this study, *Escherichia-Shigella* showed significant positive correlations with serum TNF-α, ROS, MDA and TC levels, inferring that low gut abundance of *Escherichia-Shigella* may cause low-level inflammation. Previous research has reported that feeding fermented feed can reduce the enrichment of *Escherichia-Shigella* in the intestines (17), consistent with this study. The decrease of *Escherichia-Shigella* abundance resulting from the addition of fermented diet can be attributed to the probiotics or products within the fermented diet adhering to intestinal epithelial cells to inhibit the attachment and invasion of pathogenic bacteria. Furthermore, lactic acid bacteria contribute to lowering intestinal pH through the secretion of organic acids such as lactic acid and acetic acid. This acidic environment inhibits the growth of pathogens. *Aerococcus* is clinically associated with endocarditis, arthritis, and urinary tract infections in both humans and pigs. The injection of *Aerococcus* significantly increased the protein expression of TNF-α, IL-1β, and IL-6 in the mammary glands of the mice (76). The low abundance of *Aerococcus* caused by fermented diet addition indicated low inflammation. In summary, the fermented diet addition can reduce the abundances of harmful bacteria and increase the abundances of beneficial bacteria in the feces of sows, thus regulating the balance of gut microbiota to improve overall intestinal health and reproductive performance of pregnant sows.

## 5 Conclusion

This study indicated that adding 15% fermented diet in the diet of sows during late pregnancy could increase litter weight, nutrient digestibility, antioxidant capacity and immune response of sows as well as regulate gut microbial balance. This research provided the crucial theoretical support for its application in improving health status and reproductive performance of late-gestation sows. The future research may focus on the preparation of fermented diets by different microbial species and its active mechanism in improving sow reproduction during the whole pregnancy and lactation periods.

## Data Availability

The original contributions presented in this study are included in this article/supplementary material, further inquiries can be directed to the corresponding authors.

## Footnotes

1
http://www.drive5.com/uparse/

2
https://sourceforge.net/projects/rdp-classifier/

3
www.majorbio.com
